# The Role of Extracellular Matrix (ECM) Adhesion Motifs in Functionalised Hydrogels

**DOI:** 10.3390/molecules28124616

**Published:** 2023-06-07

**Authors:** Anna J. Morwood, Ikhlas A. El-Karim, Susan A. Clarke, Fionnuala T. Lundy

**Affiliations:** 1 Wellcome-Wolfson Institute for Experimental Medicine, School of Medicine, Dentistry and Biomedical Sciences, Queen’s University Belfast, 97 Lisburn Road, Belfast BT9 7BL, UK; amorwood01@qub.ac.uk (A.J.M.); i.elkarim@qub.ac.uk (I.A.E.-K.); 2 Medical Biology Centre, School of Nursing and Midwifery, Queen’s University Belfast, 97 Lisburn Road, Belfast BT9 7BL, UK; s.a.clarke@qub.ac.uk

**Keywords:** biocompatibility, tissue engineering, biomimetic peptide, dental pulp, bone, lung

## Abstract

To create functional tissue engineering scaffolds, biomaterials should mimic the native extracellular matrix of the tissue to be regenerated. Simultaneously, the survival and functionality of stem cells should also be enhanced to promote tissue organisation and repair. Hydrogels, but in particular, peptide hydrogels, are an emerging class of biocompatible scaffolds which act as promising self-assembling biomaterials for tissue engineering and regenerative therapies, ranging from articular cartilage regeneration at joint defects, to regenerative spinal cord injury following trauma. To enhance hydrogel biocompatibility, it has become imperative to consider the native microenvironment of the site for regeneration, where the use of functionalised hydrogels with extracellular matrix adhesion motifs has become a novel, emerging theme. In this review, we will introduce hydrogels in the context of tissue engineering, provide insight into the complexity of the extracellular matrix, investigate specific adhesion motifs that have been used to generate functionalised hydrogels and outline their potential applications in a regenerative medicine setting. It is anticipated that by conducting this review, we will provide greater insight into functionalised hydrogels, which may help translate their use towards therapeutic roles.

## 1. Introduction

The functionalisation of hydrogels with specific adhesion motifs is a well-recognised contribution to their biocompatibility. An emerging theme for hydrogels utilised in tissue engineering and regenerative medicine purposes, is for their adhesion motifs to mimic the extracellular matrix at the tissue engineering site; however, to date, a comprehensive review of extracellular matrix (ECM) components and naturally occurring adhesion motifs is lacking. In this review, we introduce hydrogels for tissue engineering purposes, describe the various components of the ECM (including those from different anatomical sites) and critically appraise specific adhesion motifs that have been exploited in hydrogel research. By reviewing the literature in this emerging research field, we aim to improve understanding of hydrogel functionality and expedite the translation of biomimetic hydrogels to therapeutic use.

### 1.1. Tissue Engineering and Regenerative Medicine

Tissue engineering encompasses three main elements in the form of cells, biomaterials and biophysical factors, which function collectively to maintain, restore or replace various tissues [1]. These fundamental elements are identified as the triad of tissue engineering, where successful healing relies on all elements working in tandem to promote sufficient regeneration [2]. The process of regeneration is driven by a complex array of biological signals produced by the ECM, allowing attraction, proliferation and differentiation of stem cells towards an appropriate lineage, depending on the tissue in question [2]. For tissue engineering to be successful, cells must be efficiently transplanted into the site for regeneration. Cells must be able to survive at the regeneration site within an appropriate scaffold for a sufficient period of time, to allow the release of growth factors and/or other molecules that may drive tissue regeneration [3]. The need for new bioactive materials has driven forward research in the fields of tissue engineering and regenerative medicine to develop biomaterials that mimic the natural ECM. In this review, we will focus on adhesion motifs that occur naturally in the ECM and their potential use in tissue engineering biomaterials (principally hydrogels). Before focusing on the adhesion motifs used in vitro, some background to hydrogels and the role of the ECM in vivo is discussed.

### 1.2. Hydrogels as Sophisticated, Biomimetic Materials

Hydrogels are three-dimensional (3D), crosslinked, fibre networks composed of a water soluble polymer [4]. They have desirable physiochemical properties which contribute to their many biomedical applications, such as drug delivery, implant coatings and tissue engineering [5]. Hydrogels are formed through physical or chemical crosslinking of 3D fibre networks [6]. Physical crosslinking connects fibres through hydrogen bonding, electrostatic interactions, or hydrophobic interactions to form secondary alpha (α) helical or beta (β) sheet structures [7]. Chemically crosslinked hydrogels contain covalent bonds induced by polymerisation reactions such as enzyme polymerisation and photo-polymerisation [7]. Important features of hydrogels include their biocompatibility, biomimetic nature towards the ECM and high water content [8]. Mechanical properties such as mesh size and swelling, along with degradation rate, play important roles in biomaterial stability [9].

Hydrogels are excellent cell culture matrices due to their ability to provide a 3D culture environment rather than a 2D environment, enhancing their ability to accurately mimic the ECM [10]. They can be prepared from ECM materials such as collagen or fibronectin to produce hydrogels with enhanced biocompatibility [10]. Additionally, the physical properties of hydrogels themselves can influence how the material resembles the ECM. For instance, mesh size and molecular porosity control the passage of nutrients and waste through the hydrogel matrix, resembling how these components travel through the native ECM [9]. Crosslinked hydrogels can result in biomimetic properties which resemble that of soft tissues, such as viscoelasticity and interstitial flow [11]. Hydrogels also contain large volumes of water, much like the ECM, and are often described as water-swollen polymer networks [12]. The hydrophilic nature of hydrogels is associated with the presence of polymeric backbone motifs containing carboxyl, amine and hydroxyl groups [13].

#### 1.2.1. Synthetic Hydrogels

As the classification suggests, synthetic hydrogels are chemically synthesised and are thus manufactured via highly reproducible methods [14]. They also have the advantage of having “tuneable” properties. For example, the mechanical properties of hydrogels such as stiffness and/or crosslinking can be specifically altered or tuned to mimic the various ECM microenvironments of different tissues. Mechanical properties such as these also have a profound effect on the mechanotransduction of cells within a hydrogel [8], contributing to the conversion of physical cues into biochemical signals, leading to enhanced cell behaviours such as spreading and migration [9]. Molecular cues can also be incorporated within the chemical formula, such as cell adhesion motifs, to improve cell adhesion performance. The challenges associated with synthetic hydrogels should also be considered, where there is a possibility that the biomaterial itself or any by-products produced may have a toxic effect, resulting in a loss of biocompatibility.

##### 1.2.1.1. Polymer-Based Hydrogels

A common example of a synthetic hydrogel-forming polymer is Poly(lactic-co-glycolic acid) (PLGA), which consists of glycolic acid and lactic acid monomers connected by ester bonds [15]. This FDA approved polymer is advantageous for tissue engineering applications due to its biocompatibility and controllable degradation rate [15]. In 2006, PLGA scaffolds coupled with adipose-derived stem cells were investigated for the generation of muscle tissue [16]. PLGA has also been shown to promote cell viability and differentiation, demonstrating its suitability as a cell culture scaffold [17].

Poly(ethylene glycol) (PEG) is another polymer commonly used for hydrogel formation, with a unique ability to resist protein absorption [15]. PEG scaffolds are normally polymerised using chemical or photoinitiators. These scaffolds have been noted for their suitability as candidates for stem-cell-based tissue engineering in tissues such as liver, heart and nerve. Extensive research has also investigated the use of PEG scaffolds seeded with stem cells to generate bone and cartilage [18,19].

##### 1.2.1.2. Peptide-Based Hydrogels

Peptide hydrogels have emerged as a novel class of hydrogels, which bridge the gap between natural and synthetic hydrogels. They can be synthesised easily and reproducibly using laboratory manufactured peptides, and are also deemed to be highly versatile, as their amino acid sequences can be modified to promote advantageous functional traits [20]. Peptide-based hydrogels self-assemble in solution through sequence complementarity to form 3D hydrogels [21], with hydrogen bonding, electrostatic interactions and hydrophobic interactions contributing to the self-assembly process [22]. Generation of secondary structures such as alpha helices or beta sheets can be enhanced by the ratios of certain amino acids in the peptide sequence. The resulting hydrogels display beneficial characteristics such as a biocompatible nature and an ability to mimic the structural characteristics of the native ECM [8].

Hydrogels based on self-assembling peptides are highly versatile and bridge the gap between natural and synthetic hydrogels [23]. Generally, self-assembling peptides are synthesised using fluorenylmethoxycarbonyl (Fmoc) solid phase synthesis, which are between 15–25 amino acids in length. However, some can be less than seven amino acids, which are often termed ultrashort peptides [24]. Self-assembling peptides form hydrogels through a sol–gel transition mechanism, eliminating the need to use crosslinkers. The resulting self-assembling peptide hydrogels have a high-water content (>95%) and a nanofibrous network structure, suitable for cell encapsulation and cell culture applications [23]. The majority of self-assembling peptide hydrogels are made up of L-amino acids which are located and metabolised naturally by the body, increasing biocompatibility and reducing immunogenicity or inflammatory reactions. In addition to biocompatibility, self-assembling peptide hydrogels have shear-thinning properties that allow them to recover bulk properties following shear stress, making them ideal candidates for creating tissue engineering therapies that are less invasive for patients [23].

#### 1.2.2. Natural Hydrogels

Natural hydrogels are normally derived from naturally occurring ECM components derived from plants and animals. As a result, they are generally biocompatible, promote cell adhesion and have the capacity to hold large volumes of water, creating viscoelastic properties which resemble soft tissues [14]. However, purification of proteins from natural sources has the disadvantage of batch-to-batch variability and the potential for adverse immunogenic responses [14] and/or low mechanical strength [25]. Variability in natural hydrogels can result in diversity in their mechanical characteristics and their ability to direct differentiation [21]. Notwithstanding these limitations, protein-based hydrogels and polysaccharide-based hydrogels have emerged to play a key role in providing a biocompatible, biomimetic environment for cells to inhabit successfully.

##### 1.2.2.1. Protein-Based Hydrogels

Proteins have many desirable functional and structural characteristics, which contribute to their ability to form hydrogels, including biocompatibility and biodegradability [26]. To create a gel network, protein aggregation or physical crosslinking acts as the main mechanism contributing to protein gelation. The resulting network can sustain large volumes of water, and is stabilized by the presence of non-covalent crosslinking, such as hydrophobic interactions, van der Waals forces, electrostatic interactions and hydrogen bonding. Properties of the protein-based hydrogel such as bioactivity and hydrophobicity are dictated by the primary amino acid sequence of the protein itself [26].

Collagen is a naturally derived protein found in skin, bone and tendons. It is commonly used to create biocompatible hydrogels, providing structural support and promotion of stem cell differentiation [27]. Fibrin is another natural protein derived from fibrinogen in human and animal blood. Its ability to form a fibrous network during clot formation makes it an attractive protein for tissue engineering and wound healing applications [28]. Ryu et al. [29] reported that regeneration at infarction sites was more significant in the presence of a fibrin matrix. [29].

##### 1.2.2.2. Polysaccharide-Based Hydrogels

Polysaccharides can be derived from animals, plants, microbes or algae, and have applications in many fields such as pharmaceutics and matrix synthesis for cell culture [30]. Polysaccharide-based hydrogels can retain large volumes of water and the molecular interactions that make up these hydrogels are hydrophobic interactions, hydrogen bonding and ionic crosslinking [30]. Both alginate and chitosan are polysaccharides that can be fabricated into hydrogels. Alginate is derived from the cell walls of brown algae and has been widely used in tissue engineering and drug delivery applications [31]. Chitosan is a linear polysaccharide derived from chitin found in crustaceans, where its degree of deacetylation dictates its rheological properties [30]. The main advantages of chitosan hydrogels stem from its biocompatibility, low cost of production and antimicrobial properties [30].

#### 1.2.3. Hydrogels as “Smart” Biomaterials

The ability of hydrogels to respond to changes in their external environment has been exploited in so-called “Smart” hydrogels. Numerous stimuli such as pH, temperature, chemicals, light and electric fields induce a chemical switch within the hydrogel, causing it to swell or undergo volumetric change [32]. Other parameters such as charge [33] and degree of crosslinking can have an effect on the ability of hydrogels to absorb water [34]. In line with the tunable nature of hydrogels, swelling behaviour can also be designed to react to various stimuli experienced within the body, resulting in a 3D hydrogel matrix of desired volume for a specific application.

##### 1.2.3.1. pH-Sensitive Hydrogels

pH-responsive hydrogels are a subgroup of stimuli-sensitive matrices that can respond to changes in environmental pH [32]. These systems are particularly translatable to a range of biomedical applications, as many anatomical sites for tissue engineering can be susceptible to changes in pH, sometimes as a result of the presence of disease [32], inflamed tissues or wounds [35]. When hydrogels experience a change in pH, volumetric alterations occur, which promotes reversible and repeatable expansion of the gel [36]. Swelling is associated with charge carrying groups in the hydrogel [37]. An ion concentration is established, and osmotic pressure establishes on the surface of the hydrogel, causing ions to move in or out of the gel. Cationic hydrogels, e.g., chitosan, swell at low pH following the acquisition of protons to the charge carrying groups. The positively charged domains then cause repulsion, leading to swelling. Anionic hydrogels, e.g., carboxymethyl chitosan, swell at higher pH levels following the removal of protons from the pendant groups. The negatively charged domains cause repulsion, leading to swelling [37]. pH-sensitive hydrogels have been used as drug-delivery vehicles for releasing therapeutic agents, triggered by a change in pH [38].

##### 1.2.3.2. Light-Sensitive Hydrogels

The most common light sources used to trigger photo-sensitive hydrogel response are ultraviolet (UV), visible light and near-infrared light [39]. The presence of a photoreactive moiety, normally a photochromic chromophore, within the hydrogel polymeric network generates a response to the optical signal [40]. There are three mechanisms by which hydrogels can respond to light: (1) photo-sensitive molecules within the hydrogel convert light energy to heat, which increases the temperature of the material. When the hydrogel reaches a phase transition temperature, it becomes responsive; (2) following exposure to light, photosensitive molecules cause ionization inside the hydrogel, resulting in the generation of ions and a light response; (3) the introduction of chromophoric molecules to the polymer can cause changes in their dipole moment and their overall structure, following light exposure [40]. Light-sensitive hydrogels have shown applications for the release of small molecules for tissue engineering and wound healing applications [41], alongside drug delivery to tissues [42].

##### 1.2.3.3. Enzyme-Responsive Hydrogels

Enzyme-catalysed reactions can induce hydrogel responses, and this technique is being utilised for many biomedical applications [43]. Three requirements exist which enable hydrogels to respond to enzyme catalysis: (1) the hydrogels must include recognition elements or substrates specific to the enzyme of interest; (2) the substrates must be easily accessible to the enzymes; (3) the enzyme-substrate reaction must result in changes to the properties of the hydrogel, such as degradation [43]. Overall, enzyme-responsive systems are highly advantageous, as endogenous enzymes can be used to regulate the release of various therapeutic agents contained within the hydrogel, ensuring that their release occurs only in the presence of specific enzymes [44]. 

##### 1.2.3.4. Mechanically Sensitive Hydrogels

Hydrogels can be designed to contain surfactants which act as promoters of gelation [45]. The surfactants can cause the formation of aggregates, often driven by the presence of van der Waals forces. The resulting hydrogels have an entangled network which can trap water molecules. As outlined in Section 1.2.3.6, temperature-sensitive hydrogels can also change their mechanical properties based on their responses to temperature.

##### 1.2.3.5. Electrosensitive Hydrogels

Hydrogels that can respond to an electrical stimulus undergo a conformational change in response to an applied external electrical field [46]. Many synthetic copolymers have been reported with electrosensitive applications such as poly (vinyl alcohol) (PVA). Natural polyelectrolytes such as proteins and polysaccharides also respond to an electric stimulus. The main principle involves the movement and reorganization of ions after applying an electric field, resulting in the formation of an ion concentration gradient through the solution and hydrogel, leading to swelling or shrinkage driven by osmotic pressure. Electrosensitive hydrogels contain ionizable groups within their backbone, which generate a response to the electric stimulus. Their main applications include controlled drug delivery, and fabrication into artificial muscles for biomedical application [46].

##### 1.2.3.6. Temperature-Sensitive Hydrogels

When thermosensitive hydrogels experience a change in temperature, this changes the physical or sol–gel state of the hydrogel [47]. When stimulated by temperature, molecules with sensitivity undergo a change from a dispersed micelle to an organised 3D network structure. When the polymer solution reaches a temperature which is higher or lower than the critical dissolution temperature, phase transition will occur [47]. Temperature-sensitive hydrogels have been shown to have useful applications in protein delivery and gene therapy [48].

### 1.3. Enhancing the Complexity of Biomaterials for Tissue Engineering Applications

Moving beyond the capability of the physical structure of hydrogels to guide cell behaviour, the importance of using the native 3D microenvironment to influence cell behaviour and function has become paramount in the field of tissue engineering [49]. Research has begun to focus on ways to increase the biocomplexity of tissue engineering scaffolds, through incorporating adhesion sites or sites susceptible to proteolytic degradation [49]. When this notion was first considered, decellularized natural scaffolds were mainly used to investigate cellular behaviour. These matrices were considered to be the gold standard for organ regeneration, as they contained no native cellular components but maintained original ECM architecture and vascularity [50]. However, these materials possessed multiple limitations, whereby purification techniques caused damage to the structure of the native ECM and had considerable batch-to-batch variation in scaffold composition. Immunogenic responses often resulted from the purified materials, limiting their use for in vivo analysis [49]. Consequently, tissue engineers have begun to utilise and develop novel synthetic materials that do not possess the unpredictable variation associated with natural, decellularized scaffolds [49]. These synthetic materials can also be modified to mimic features of the native ECM in a bottom-up approach, where any scaffold is seen as a blank canvas on which scientists can add desirable features, such as motifs for adhesion or migration [51].

As most synthetic materials lack any natural promotion of cellular behaviours such as adhesion and spreading, chemical modification to promote cell adhesion is the most common biomimetic modification in the design of tissue engineering constructs. In early studies, this was achieved by coating scaffolds with ECM proteins such as fibronectin or laminin, which were known to promote adhesive properties [49]. However, further research has revealed that ECM proteins perform this key cellular behaviour through conserved amino acid sequences such as RGD or IKVAV [51]. This finding has resulted in extensive research being undertaken to develop biomimetic materials using these short peptide sequences [51], rather than the full-length proteins.

## 2. ECM Components That Contribute to Tissue Engineering Strategies

The ECM is an interconnected, 3D network of non-cellular components that form a porous, physical scaffold within which cells can adhere and embed [52]. The architecture of the ECM provides structural stability for cells, whilst allowing nutrients, oxygen, growth factors and waste products to diffuse freely [53]. Although the primary components of the ECM are water, proteins and polysaccharides, each tissue possesses a highly specific ECM composition and topography, due to biochemical and biophysical crosstalk between tissue-specific cells and the surrounding dynamic environment [54]. This crosstalk results in constant remodelling of the ECM by both enzymatic and non-enzymatic actions [55]. Despite the complex nature of any unique ECM, its architecture remains highly ordered and organised, reflecting the significance of biochemical and biophysical properties for directing the behaviour of tissue-specific cells [54].

In addition to providing a physical scaffold for cells, the ECM is responsible for initiating essential biochemical and biomechanical signals needed for tissue development, homeostasis and fundamental cell processes [56]. This is made possible as the ECM adopts a bidirectional relationship with cells, where information is passed in a reciprocal fashion from ECM to cells through specialised receptors [55], which will be discussed in Section 4.1. Within the established cellular microenvironment, signalling cascades are coordinated by intrinsic and extrinsic factors that direct cellular processes such as differentiation [57]. These factors play a crucial role in directing the cell phenotype, with changes in this microenvironment interaction causing variations in gene expression and cell behaviour [10]. In order to control the myriad of biochemical and biophysical cues presented within any microenvironment, the ECM can act as a reservoir for these bioactive molecules. Through storing and isolating particular growth factors and cytokines, concentration gradients can be established and maintained, leading to spatial and temporal regulation of molecule bioavailability [56].

The ECM is a hydrated composite containing a “core matrisome” of around 300 proteins [56], belonging to three main families of molecular effectors: (1) insoluble hydrated macromolecules, examples of these include (a) fibrillar proteins such as collagen, (b) non-collagenous glycoproteins such as laminin or fibronectin and (c) hydrophilic proteoglycans; (2) soluble macromolecules such as growth factors and cytokines; (3) proteins resident on the surface of cells [56]. Interactions between the ECM and the molecular effectors outlined above will modulate cell behaviour [11], and these merit individual discussion to more fully describe their potential roles as adhesion motifs later in this review.

The molecules that make up the ECM of specific tissues and how they are arranged ultimately determine the final ECM structure and organisation. This resulting ECM framework constitutes distinct features that aim to mirror and promote the functional capacity of the tissue in question [54].

### 2.1. Collagens

**Figure 1:**
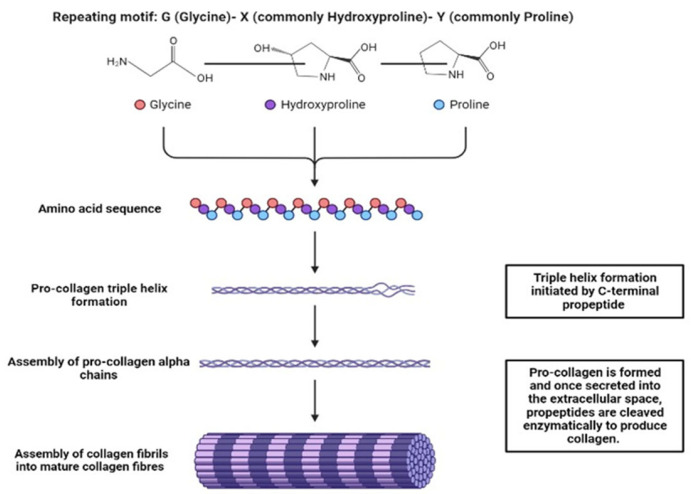
The characteristic structural feature of collagen is its triple helix- a tight right- handed helix of three α-chains. Each chain contains one or more regions encompassing the repeating amino acid motif Gly-X-Y, where X and Y can be any amino acid, but are commonly hydroxyproline and proline, respectively. Triple helix formation is initiated intracellularly by the C-terminal propeptide. Procollagen is secreted from the cell and converted to tropocollagen in the extracellular space by the removal of N and C terminal propeptides via enzyme cleavage. Crosslink formation between tropocollagen fibrils generates collagen fibre. Created with BioRender.com.

Collagens are the predominant fibrous proteins in the ECM of the majority of tissues. Functionally, they provide structural integrity and support to the ECM. Additionally, they promote tensile strength through the presence of a triple helix structure (Figure 1), and other cellular processes such as adhesion [55], which is facilitated by the presence of adhesion motifs such as the peptide sequences GFOGER and DGEA. In total there are 28 different collagen types [56] and within any tissue a heterogenous combination will exist, although one type will commonly dominate [55]. Collagen contains three polypeptide chains that form a characteristic triple helical structure [58], which can further assemble into complexes of a higher-order arrangement, such as fibrils or structural networks, depending on the type of collagen [55]. Collagens have been classified into many groups, but there are three primary classifications (Table 1): fibril-forming collagens (collagen types I, II and III), network forming collagens (collagen type IV) and fibril-associated collagens (collagen types IX, XII) [56].

### 2.2. Fibronectin

**Figure 2:**
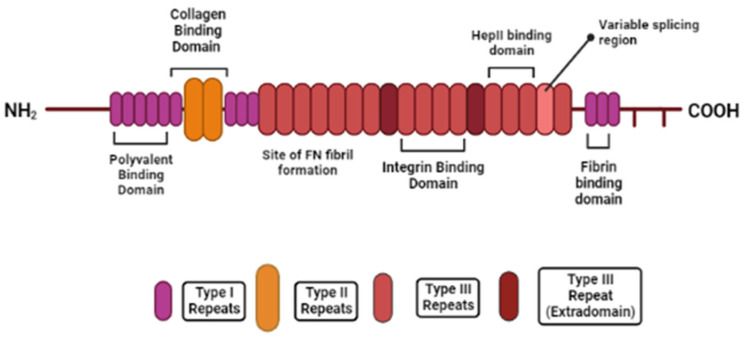
The fibronectin monomer contains several functional domains and alternative splicing regions that allow interaction of fibronectin with many other proteins of the ECM, along with cell surface receptors. These in turn promote the array of biological functions associated with the fibronectin protein. At the N-terminal region of the protein, type I repeats are responsible for producing fibronectin dimers, as well as promoting interaction with other molecules such as fibrin. Downstream of this region is the collagen-binding domain, made up of both type I and type II repeats, promoting attachment between fibronectin and type I collagen. Early type III repeats are important for fibronectin fibril formation, while other type III repeats prioritise the interaction of fibronectin with cell surface receptors, e.g., the integrin-binding domain which contains the RGD motif. Binding sites for other cell surface receptors can also be found within specific regions, such as the HepII C-terminal domain [60]. The fibronectin C-terminus acts as a site for antiparallel disulphide bonds during the formation of the fibronectin dimer [54]. Created with BioRender.com.

Fibronectin is a fibrous ECM glycoprotein found ubiquitously in all tissues [59]. The main functional roles of fibronectin are to provide organisation to the interstitial matrix [55] and to mediate essential attachment and migration of cells by acting as a “biological glue” [56]. The fibronectin monomer consists of three different repeats—type I, type II and type III—all of which have varying structures (Figure 2). Following secretion as a dimer, disulphide bonds link the fibronectin subunit at the C-terminus [56] but it can also assemble into fibrillar structures through cell-mediated mechanisms. Additionally, disulphide bonds are also found between each type I and type II repeat to enable a folded, tertiary structure. Each repeating module contains binding motifs, such as RGD [54], that are capable of promoting the wide variety of functions and binding interactions associated with the fibronectin molecule [60]. The molecule can also fold through ionic interactions, but conversely return to its original dimer position, to promote the availability of specific binding sites for other fibronectin molecules or cell surface receptors to bind [54].

### 2.3. Laminins

**Figure 3:**
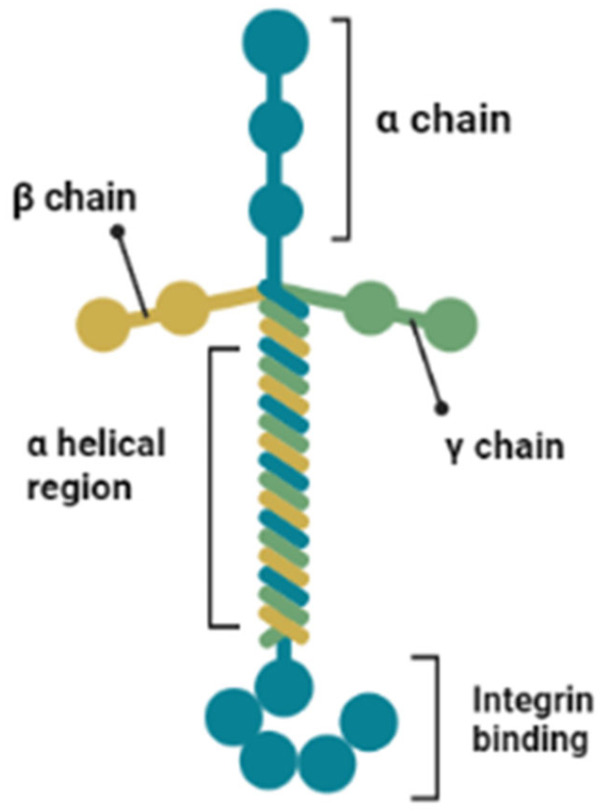
Cruciform/cross structure of laminin containing α, β and γ chains. This formation is maintained through the presence of an α helical region. Created with BioRender.com.

Laminins are characterised as cell adhesion molecules [61] within a multidisciplinary family of around 20 glycoproteins. They often crosslink and are associated with the type IV collagen network within basement membranes. The resulting self-assembled network structure maintains close connections with cells, promoting crucial interactions with cell surface receptors [56]. Laminins are also important for many biological functions, including the early stages of embryonic development and organogenesis [61]. All laminins are heterotrimers, comprising three gene products: α, β and gamma (γ) chains [62]. Structurally, these chains can form either a cruciform (cross), Y-shaped (three armed) or rod-shaped (single arm) unit, aided by the presence of a triple-helical coiled domain in the centre of each chain [54]. Similar to fibronectin, laminins consist of different domains, namely globular, laminin-type epidermal growth factor (EGF)-like repeats and α helical regions (Figure 3). These domains allow for interaction with other ECM proteins and the cell surface. Thus far, five α, four β and three γ chains have been identified, which associate to form a total of 16 distinct laminin heterotrimers [54].

### 2.4. Elastin

**Figure 4:**
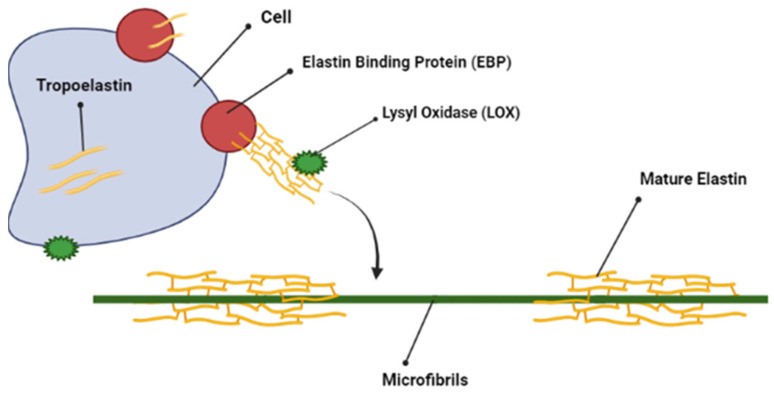
Tropoelastin contains alternate hydrophobic and hydrophilic regions. Each region contains specific motifs that are involved in elastin fibre assembly induced by the enzymatic action of lysyl oxidase (LOX), producing mature elastin. Tropoelastin is synthesised inside the cell, where it binds to the elastin-binding protein (EBP), forming a transmembrane elastin receptor. This protein transports the tropoelastin to microfibrils which are found outside the cell, where the process of elastic fibre assembly starts. Tropoelastin is released from the EBP through galactosugar microfibril binding. Subsequently, lysine residues in tropoelastin undergo deamination by the LOX enzyme through oxidation. This forms crosslinking sites that result in the formation of mature elastin. Created with BioRender.com.

The specific components of the ECM that are accountable for elasticity and expandability are the elastic fibres, which play crucial biological functions in many organs and tissue types such as arteries, skin and tendons [63]. Elastic fibres contain two structurally distinct components: a group of aligned microfibrils based on fibrin, and a dense network of crosslinked elastin, which normally contributes around 90% of total fibre content [63]. The microfibril component of the elastic fibres are usually 10–12 nanometres (nm) in width, which provides tissues with extensive elasticity. Elastin is an insoluble, 70 kilodalton (kDa) protein component derived from the more soluble precursor tropoelastin [63]. Elastin primarily serves to promote recoil in tissues that are exposed to repeated stretching during normal tissue function [55]. It also has excellent durability attributed to its ability to resist proteolytic degradation [63]. The formation of mature elastin results from a multistep process involving intracellularly synthesised tropoelastin, elastin-binding proteins (EBP) and microfibrils. Elastin fibre assembly is then initiated, which eventually results in the formation of the final elastin product (Figure 4) [64].

### 2.5. Proteoglycans and Glycosaminoglycans

Proteoglycans are expressed ubiquitously in all ECMs and consist of a main protein core with one or more covalently attached, negatively charged glycosaminoglycans.

Proteoglycans play a central role in the ECM, performing many structural and biological functions, including hydration of the ECM, homeostasis, structural organisation and regulating cell signalling cascades, such as those required for adhesion, migration and proliferation [63]. Glycosaminoglycans form an integral part of proteoglycan structure as unbranched, linear, repeating disaccharide chains [65], which include heparan sulfate, heparin, chondroitin sulfate, keratan sulfate, dermatan sulfate and hyaluronic acid. These 6 distinct glycosaminoglycan groups result from differences in structure of the disaccharide unit, such as their composition, size or variations in the position of sulfonylation sites. This element of variation explains the structural and functional diversity attributed to proteoglycans [63], which can be classified into four main groups: intracellular proteoglycans, cell surface proteoglycans, pericellular proteoglycans and extracellular proteoglycans (Table 2), all of which are bound by varying glycosaminoglycan chains [66].

## 3. Composition of ECMs from Various Tissues 

In previous sections of this review, the importance and fundamental properties of the ECM have been highlighted. To begin considering how the ECM can be utilised to create biomimetic materials for regeneration, it is important to consider how the exact composition of the ECM varies from tissue to tissue. The organisation, internal makeup and characteristics of any given matrix ultimately depends on the specific tissue of origin. In a review of this size, it is not possible to elaborate on the ECM from a large number of tissue types. In the sections that follow, the composition of the ECM in three selected tissues, dental pulp, pulmonary tissue and bone tissue, with varying functionalities and mechanical stresses, will be discussed.

### 3.1. Dental Pulp ECM

The dental pulp is a soft, vascular, connective tissue found in the centre of the tooth, which provides sensory function, nutrition [67] and reparative capacity [68]. The ECM formed by the dental pulp is highly hydrated and encompasses a range of cell types, including fibroblasts, mesenchymal stem cells (MSCs), macrophages and lymphocytes, along with the factors they secrete. Pulp fibroblasts secrete a complex ECM that varies significantly to other soft connective tissues, although it constitutes some of the most significant ECM proteins such as collagen, fibronectin, proteoglycans and glycosaminoglycans [68]. There are four main subtypes of collagen found within the dental pulp: type I (56%); type III (41%); type V (2%) and type VI (0.5%), where type I and III are most abundant [68]. Collagen fibrils (especially type I) usually form long, thick, parallel sheets throughout pulp tissue, which provides crucial structural support to the matrix [69]. The main non-collagenous protein found within the dental pulp is fibronectin, although its specific abundance in the dental pulp has not been reported. Despite this, it is known that fibronectin tends to form a complex with type III collagen in a 1:1 ratio [68]. Proteoglycans and glycosaminoglycans contribute to the dental pulp ECM by providing tissue turgor and facilitating cell–cell interactions [69]. The most common proteoglycans of the dental pulp are decorin, biglycan and versican, with the following glycosaminoglycan modifications also being present: 4-and-6 chondroitin sulfate (60%); dermatan sulfate (34%); and keratan sulfate (2%) [68]. The protein component elastin has a structural role in the blood vessels of the dental pulp, but it does not form part of the ECM itself [69].

### 3.2. Pulmonary ECM

The pulmonary ECM contains a dynamic array of components, such as fibrous proteins, glycoproteins, glycosaminoglycans and proteoglycans [70]. A wide range of cell types also promote and maintain tissue architecture, including fibroblasts, smooth muscle cells and epithelial cells [71]. Within the pulmonary system, two fundamental structures exist in the form of basement membranes and interstitial matrices [72]. The basement membrane predominantly contains components such as collagen, laminins, chondroitin sulfate proteoglycans and fibronectin. Conversely, the interstitial matrix hosts a meshwork of components such as elastin, versican, fibronectin, vitronectin and decorin [71]. Amidst the molecular complexity of the pulmonary ECM, collagen is the most abundant contributor to the protein matrisome. Fibrillar collagens contribute to the overall structure and integrity of the lung, with collagen subtypes I and III promoting structural integrity in the alveolar walls and subtype IV being mainly located in basement membranes. Elastic fibres are responsible for providing an element of stretch and recoil to the lungs [72].

### 3.3. Bone ECM

The ECM of bone contains both organic and inorganic compounds, which make up 40% and 60% of the total matrix, respectively [73]. The organic component consists of type I collagen (90%), along with non-collagenous proteins (10%) [73], while the inorganic component of the matrix consists primarily of hydroxyapatite and trace elements such as magnesium and zinc [74]. Type I, type III and type V collagens are abundant in the organic component of the ECM of bone. Like most other ECMs, collagen in bone tissue serves to provide mechanical support and forms a protective network for bone cells to reside. Type I collagen forms triple helical structures, which can be assembled to form higher-order fibril structures (Figure 1). Although types III and V collagen are present in smaller amounts, they also play a crucial role in regulating the formation of collagen fibres [73].

In addition to collagen, non-collagenous proteins are arranged into an ordered and specific network [75] in the ECM of bone. These include proteoglycans or small leucine-rich proteoglycans (SLRPs), such as decorin or keratocan. These play an important role in regulating bone homeostasis and controlling cellular behaviours by interacting with cell surface receptors to promote cell proliferation, osteogenesis, mineral deposition and bone remodelling. Osteocalcin, a γ-carboxyglutamic acid-containing protein is an additional important organic component of bone and is a marker of bone formation. Finally, Small Integrin-Binding ligand N-linked Glycoproteins (SIBLINGS) are a group of glycophosphoproteins, including bone sialoprotein (BSP) and osteopontin, which are found in the organic matrix of bone [73].

## 4. Cell Adhesion Molecules

**Figure 5:**
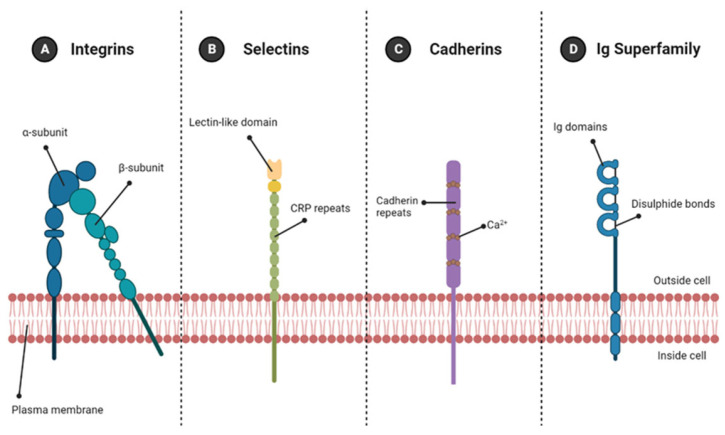
The four distinct families of cell adhesion molecules, which facilitate cell-to-cell binding or binding of cells to the ECM through adhesion. Adapted from [77] under Creative Commons CC BY license. Created with BioRender.com.

Interaction between the ECM and cells resident within it depends on the presence of cell adhesion proteins, expressed on the surface of cells, which act as matrix receptors and provide a link between the ECM and cell cytoskeleton [76]. There are four major families of cell adhesion molecules: integrins, immunoglobulin super family, cadherins and selectins (Figure 5).

### 4.1. Cell-ECM Adhesion Molecules

#### Integrins

Integrins are heterodimeric, cell–matrix adhesion receptors composed of α and β subunits [56], that can bind ECM proteins such as fibronectin, collagens and laminins [76]. For example, α2β1 can bind to certain members of the collagen family, α5β1 binds to fibronectin, and αvβ3 shows more promiscuous binding to a selection of ligands including fibronectin, vitronectin and fibrinogen [78]. Both the α and β integrin subunits are membrane traversing glycoproteins with extracellular domains, single transmembrane helices and cytoplasmic regions, allowing the integrin molecule to interact successfully with the cytoskeleton. Integrins are normally sub-grouped depending on their ligand-binding affinities or on their subunit composition. Generally, the most common integrins are the β1 integrins, which are known to show a strong affinity for the major ECM proteins fibronectin, collagens and laminins [56].

Following interaction of the integrin receptor with ECM proteins, the integrin itself undergoes a conformational change [56]. As a result, focal complex proteins such as focal adhesion kinase (FAK) and integrin-linked kinase (ILK) bind to newly exposed integrin cytoplasmic domains, forming a focal adhesion complex and initiating clustering of integrin molecules at the membrane site. This leads to activation of specific intracellular signalling cascades such as the mitogen-activated protein kinase (MAPK) pathway, leading to the regulation of essential cellular processes such as proliferation, differentiation, polarity and gene expression in what is known as an “outside-in” signalling process [56]. It is important to note that signals from intracellular proteins can also alter integrin activation and shape, changing their ligand-binding capacity in an “inside-out” signalling process. Thus, integrins are bi-directional signalling units that transmit information and allow connections between both intracellular and extracellular milieu [56]. 

### 4.2. Cell–Cell Adhesion Molecules

#### 4.2.1. Immunoglobulin Superfamily

The immunoglobulin superfamily (IgSF) has over 765 diverse members and is one of the largest groups of proteins found in the human body [79]. The most common members of the IgSF are T cell receptor complex proteins, major histocompatibility complex class 1 and 2 molecules, virus receptors and cell surface glycoproteins [79]. Almost all members of the superfamily are classed as single-pass transmembrane proteins, comprising an extracellular domain with one or multiple Ig-resembling repeats and intracellular domains of varying length [80]. Other members can be tethered to the plasma membrane of the cell through a glycosylphosphatidylinositol (GPI) unit. IgSF components, especially Ig domains, can promote both homophilic and heterophilic cell adhesion, while specialised linker proteins couple the IgSF component to cytoskeletal regions [80]. One of the major functions of the IgSF is to promote cell–cell adhesion. In particular, a subgroup of the IgSF that executes this role is the nectin family, which promotes homophilic or heterophilic cell–cell attachment in various tissues [81].

#### 4.2.2. Cadherins

Hyafil and Peyrieras first reported cadherin in the 1980s using the name “Uvomorulin” in mice [82]. Since then, numerous subtypes have been identified in different species, resulting in more than 100 members of the family now being recognised. The cadherins are transmembrane glycoproteins that promote cell–cell adhesion, which is highly dependent on calcium ion association [82]. Cells make use of cadherins not just for cell–cell adhesion during morphogenesis, but also to regulate cell signalling and control the fate, polarity and proliferation of cells [83]. The activation of cadherins mainly occurs due to the formation of transbonds at the contact site between two cells. Once this connection has been formed, cadherins regulate cell–cell attachment in one of three ways; (1) reducing local tension; (2) actomyosin signalling; and (3) establishment of mechanical coupling [83]. The cadherin classification system includes several subtypes: type I classical cadherins such as E-cadherin, N-cadherin and P-cadherin, type II classical cadherins such as VE-cadherin and OB-cadherin, desmosomal cadherins such as the desmocollins, seven-pass transmembrane cadherins such as flamingo, FAT atypical cadherin (FAT) and dachsous group cadherins such as FAT cadherin and protocadherins such as C-protocadherin (C-Pcdhs) [82]. Despite their many classes, all cadherins are composed of three main domains including an extracellular domain, a single-pass transmembrane domain and a cytoplasmic domain [82]. The presence of a peptide cadherin domain containing around 110 amino acids also serves to facilitate homophilic, calcium dependent interactions between different cadherin molecules [84].

#### 4.2.3. Selectins

Selectins are a family of cell adhesion molecules found predominantly in the vascular system, and are described as transmembrane, calcium (Ca^2+^) dependent lectins [85]. The three main types are L-selectin, E-selectin and P-selectin, which are specialised in leukocyte capture and rolling on the surface of vascular cells [86]. L-selectin is found expressed on the surface of leucocytes, P-selectin is expressed by megakaryocytes and endothelial cells, and E-selectin is expressed on the surface of venular endothelial cells [85]. All selectins contain a similar structure, encompassing an N-terminal C-type (Ca^2+^) lectin domain, an EGF domain, 2–9 sushi domains, a transmembrane domain and a cytoplasmic domain [87].

## 5. ECM-Derived Peptide Sequences and Their Use in Biomimetic Hydrogel Functionalisation

Cell adhesion molecules that bind to the ECM do so by short peptide recognition sequences in ECM proteins. These recognition sites are found at sequence specific peptide regions of the protein, and characterising these regions is considered a challenging task due to the supramolecular complexity of most ECM proteins [88]. Despite this, modifying biomaterials with these short peptides allows important ECM characteristics to be incorporated into an environment which otherwise may exert little to no biomimetic potential [49]. In this section, the most relevant peptide sequences previously identified in ECM proteins, such as collagen, fibronectin, laminin and elastin will be discussed. The use of these peptide sequences either alone or in combination could prove to be a powerful tool for creating biomimetic materials that replicate the ECM, particularly in the context of hydrogel functionalisation.

### 5.1. Fibronectin: RGD Motif

The tri-amino acid sequence RGD is the most extensively researched peptide adhesion motif. RGD serves as the main integrin-binding domain (residues 1615–1617 of fibronectin), and is found in many other ECM proteins such as vitronectin, collagen and bone sialoprotein [78]. In 1984, RGD motifs were first discovered as cell adhesion molecules, and the tripeptide sequence was identified as the minimal essential cell adhesion sequence of the ECM protein fibronectin [89]. The tendency for RGD to bind to an array of integrin receptors is thought to stem from the conformation of the loop containing RGD and the respective flanking amino acids [89]. In the literature, the relationship between RGD motifs within larger ECM proteins and cell attachment is evident. It has also been suggested that even small, synthetic peptides that contain the RGD sequence can promote cell attachment in a similar way to the much larger parent molecule fibronectin, highlighting the extent of its efficacy [90].

Numerous groups have sought to imitate the roles of the ECM by functionalising hydrogels with the RGD motif. Human periosteum-derived cells (hPDCs) were encapsulated in PEG hydrogels functionalised with RGD, and results demonstrated the potential use of these hydrogels in articular cartilage regeneration to treat joint surface defects [91]. The hydrogels functionalised with RGD gave an average cell viability of approximately 85%, whereas cells cultured in hydrogels without RGD had viability of approximately 65%, decreasing to 40% after 4 weeks in culture [91]. A similar trend has also been identified elsewhere in the literature, where inclusion of the RGD peptide motif in various hydrogels can sustain a high cell survival rate [92,93]. Corresponding to increased cell proliferation, DNA content in cells grown within hydrogels containing RGD was ten-fold higher than hydrogels without RGD (*p* < 0.001) [91]. Other groups have similarly published cell viability, spreading and proliferation data which agree with the above results [94,95,96], providing evidence that RGD can be used as a biomimetic motif to replicate the adhesive characteristics of the ECM.

The ability of commercial Vitrogel hydrogels with the RGD motif (VG-RGD), or without the RGD motif (VG-3D), to support the differentiation of human adipose stem cells (hASCs) has been investigated [97]. Following encapsulation, cells were evenly distributed throughout both hydrogels with high viability, indicating the promotion of a stable environment capable of cell adhesion and long-term cell support, much like the native ECM [97]. Moreover, hydrogels functionalised with RGD showed significantly enhanced differentiation of hASCs to a chondrogenic lineage [97], which has also been reported by other groups using different hydrogel types functionalised with RGD [98]. These results suggest that addition of RGD to hydrogel matrices can favour cell differentiation towards selected lineages. In relation to cartilage regeneration, the hydrogels were shown to have the functional capacity to replicate the complex characteristics of cartilaginous ECM, providing precise control of cellular functions to drive new tissue formation [97].

### 5.2. Collagen: GFOGER Motif

The α1β1 and α2β1 integrins are known to interact with a variety of collagen subtypes. The type I collagen α2β1 integrin recognition site is reported to contain the amino acid sequence (GFOGERGVEGPOGPA), located at residues 502–516 of the α1(I) chain [99]. Further work confirmed that the N-terminal hexapeptide sequence, GFOGER, within the 15-residue amino acid sequence, is entirely responsible for providing integrin recognition (residues 506–511). It has been shown that the GFOGER sequence can promote cell adhesion through both the α1β1 and α2β1 integrin receptors [99].

The GFOGER sequence has been used extensively throughout the literature as a biomimetic motif to modify hydrogels, with the aim of controlling vital cellular processes conducted by the ECM. In a 2014 study, hMSCs were encapsulated in degradable and non-degradable PEG hydrogels, modified with the sequence GPC(GPP)_5_-GFOGER-(GPP)_5_GPC-NH2 [100]. Cell proliferation following hydrogel encapsulation was monitored using a bromodeoxyuridine assay, where proliferation was found to be four- fold higher in GFOGER-modified degradable gels compared with degradable gels without any adhesion motif (*p* = 0.017) [100]. Proliferation was also two-fold higher in degradable GFOGER hydrogels compared with RGD-modified degradable hydrogels (*p* = 0.069) [100]. The deposition of glycosaminoglycans within the hydrogels was measured both quantitatively and qualitatively as a marker for chondrogenic differentiation. The GFOGER-modified degradable hydrogels promoted the highest glycosaminoglycan content, as well as higher expression of the chondrogenic markers type II collagen and aggrecan. These results suggest that GFOGER-modified hydrogels can successfully support cell proliferation and the deposition of a chondrogenic ECM, and could be employed for MSC-based treatments of cartilage lesions [100].

A collagen mimetic peptide containing the GFOGER sequence ((GPO)_4_GFOGER(GPO)_4_GCG) has also been incorporated into PEG hydrogels and investigated for the ability to enhance chondrogenic differentiation of MSCs [101]. PEG hydrogels with the integrin specific GFOGER collagen sequence were shown to display improved cell viability and chondrogenic differentiation [101]. This study shows similar application, outcome measures and results compared with Mhanna et al. [100], the only difference being the shorter peptide length of (GPO)_4_GFOGER(GPO)_4_GCG, suggesting that it could deliver similar results. Synthetic PEG hydrogels containing the GFOGER motif have also been investigated for their ability to stimulate survival and osteogenic differentiation of human-bone-marrow-derived stem cells (hBMSCs) [102]. It was shown that GFOGER-modified hydrogels stimulated elevation of FAK phosphorylation compared with inactive GAOGER-modified hydrogel controls, suggesting that this peptide may harbour the potential to biologically mimic essential ECM cell signalling mechanisms [102]. GFOGER-modified hydrogels were shown to support cell adhesion, signalling and osteogenic reparative functions when transplanted into murine bone defects, replicating some of the properties of the ECM in vitro and in vivo [102]. 

Ha et al. [103] prepared an in situ forming polymer hydrogel, conjugated with GFOGER, named polyethylene glycol-b-polycaprolactone (GFOGER-PEG-PCL), utilising various GFOGER concentrations. The expression of β1, α2 and α11 integrins in encapsulated BMSCs was monitored as a measure of adhesive capacity. In the polymer hydrogel modified with 0.8 mM GFOGER, a strong expression of all three integrins was detected. Following 7 days culture, cell proliferation in the PEG-PCL hydrogel modified with 0.8 mM GFOGER showed a four-fold increase in DNA production, compared to controls. The ability of the hydrogel to regenerate articular cartilage and subchondral bone was also assessed. The defect treated with PEG-PCL conjugated with 0.8 mM GFOGER showed the formation of dense, integrating neo-cartilage and subchondral bone. This was compared to methoxypolyethylene glycol-b-polycaprolactone (MPEG-PCL) in the absence of GFOGER, where only peripheral tissue formation was seen with no significant density. The polymer hydrogel allowed long-term retention of BMSCs, while promoting an enhanced osteochondral regenerative capacity [103].

### 5.3. Collagen: DGEA Motif 

DGEA is a tetrapeptide sequence found between residues 232–235 and 613–616 of the type I collagen sequence and is an α2β1 integrin-binding site [104,105,106]. Staatz et al. [104] synthesised peptides to investigate the inhibition of platelet adhesion to collagen. It was shown that DGEA was the α2β1 recognition sequence [104] and that integrin-collagen interaction could be disrupted by the addition of DGEA peptides, providing evidence of the importance of this sequence in α2β1 integrin binding [107,108].

To investigate the DGEA motif as a biomimetic ECM peptide, alginate hydrogels were conjugated with DGEA and tested for their ability to promote cell adhesion, cell viability and osteogenic differentiation [106]. MSCs displayed limited cell adhesion and spreading on hydrogels modified with DGEA, compared with MSCs adhered to hydrogels modified with RGD [106]. These results correlated with cell viability data, where cells that were seeded onto RGD-modified hydrogels demonstrated metabolic activity that was significantly higher than MSCs on DGEA-modified hydrogels [106]. In this study, it was also hypothesised that hydrogels presenting DGEA motifs may promote osteogenic differentiation of MSCs. Indeed, all peptide-modified hydrogels including RGD, DGEA or both, showed a higher percentage of cellular alkaline phosphatase (ALP) activity than unmodified hydrogels [106]. Moreover, DGEA hydrogels stimulated the most significant collagen I and osteocalcin production compared to all other hydrogels tested [106]. Although MSCs demonstrated lower cell adhesion and viability on hydrogels modified with DGEA, their capacity for osteogenic differentiation was enhanced, suggesting that this peptide motif could be used to direct differentiation in the ECM.

### 5.4. Collagen: P15 Motif

P15 is a short synthetic peptide sequence (GTPGPQGIAGQRGVV) [109], derived from the α1helix domain of type I collagen at residues 947–961. It acts as a cell-binding domain of collagen by stimulating α2 integrin activation. Numerous studies have demonstrated that P15 can promote attachment for cell types such as fibroblasts and osteoblasts [110]. The P15 motif has also been reported to have applications for coating synthetic bone materials in order to promote osteoblast differentiation, cell adhesion, migration and survival. In addition, it has been linked to an increased MSC expression of ALP, which is an early indicator of osteoblastic differentiation [109].

The P15 collagen motif has been used to coat anorganic bone material (ABM), which was suspended in hyaluronate (Hy) hydrogels to mimic the ECM of bone [111].The study suggested that the ABM/P15 hydrogel matrices can act as an injectable, biomimetic material to promote bone repair. Hydrogels with (ABM/P-15/Hy) and without P15 (ABM/Hy) were tested, where osteoblast-like cells were seeded onto each hydrogel. A greater cell presence and increased cell scattering were observed on the ABM/P15/Hy surface, but only small areas of cells on ABM/Hy, suggesting the P15 peptide promotes cell attachment and migration [111]. These results were confirmed by quantitative assays whereby adherence on ABM/P15/Hy was significantly increased compared with the ABM/Hy surface (*p* < 0.05), and cell viability was double, for cells cultured on ABM/P-15/Hy [111]. Alongside fundamental cell processes, the ability for P15 to influence osteoblastic activity was considered. Following 10 days culture, ALP, bone morphogenetic protein 2 (BMP-2) and bone morphogenetic protein 7 (BMP-7) were found to have significantly higher expression levels in cells grown on ABM/P-15/Hy, compared with those grown on ABM/Hy [111]. Increased expression of osteogenic genes suggests that P15 may be an initiator of osteoblastic commitment by promoting a capacity for differentiation, much like the native ECM [111]. Even though this study incorporated the P15 motif on a material suspended within a hydrogel, promising results were still obtained in relation to enhancing biomimetic capacity. Further work should be undertaken to investigate the biomimetic traits of P15 through direct hydrogel functionalisation.

### 5.5. Fibronectin: LDV Motif

LDV is a fibronectin-derived tri-amino acid sequence, capable of promoting cell adhesion in numerous cell types, such as lymphocytes [112]. This specific adhesion site is found in the connecting segment 1(CS1) peptide within the Heparin II/III connecting segment (Hep II/IIICS) region, in which the III/connecting segment domain (III/CS domain) or variable region (V region) acts as an α4β1 recognition site [112], specifically between residues 2102–2104 and 2466–2468. LDV-containing sequences are considered to be one of the most significant cell adhesion sites found in fibronectin, alongside the RGD sequence [113].

The first incorporation of the tripeptide motif LDV within a supramolecular peptide hydrogel was reported by Cringoli et al. [114], where hydrogel composition included the self-assembling tripeptide FFL, a key initiator of supramolecular hydrogel formation, along with the bioactive motif LDV [114]. The formed hydrogel with and without the presence of LDV showed evidence of high fibroblast viability; however, cell spreading within the hydrogel was only observed in the presence of LDV [114]. Through the addition of manganese (Mn^2+^), a known co-factor for integrin binding, the cell adhesion response towards LDV increased, implying that the LDV motif was capable of successfully mimicking the ECM [114]. The addition of an integrin-β1-blocking antibody conversely hindered cell adhesion, further confirming the relationship between integrins and the LDV motif as a driver of cell adhesion. Overall, this peptide scaffold with the addition of an LDV motif can be seen as a biomimetic hydrogel capable of cell adhesion, spreading and viability through activation of β1 integrin proteins [114].

### 5.6. Laminin: IKVAV Motif

The pentapeptide sequence IKVAV is derived from the laminin α1 chain [115], found between residues 2116–2120. The N-terminal isoleucine (I) residue is considered to be vital for promoting cell adhesion activity [116]. Various synthetic peptides from the A chain region of laminin were tested for biological activity and a 19-mer peptide found above the carboxyl globule of the A chain was found to be a promoter of multiple key cellular processes, such as cell adhesion and migration. After testing shorter sequences within this peptide, the pentapeptide IKVAV was confirmed to be an active site for cell adhesion, but more specifically neurite outgrowth. Results concluded that the IKVAV sequence is a principal site within the ECM protein laminin, which serves to regulate cell behaviour [116].

The pentapeptide motif IKVAV was used to functionalise poly (lactide ethylene oxide fumarate) (PLEOF) and create a hydrogel capable of mimicking the ECM [117]. The regenerative environment created was suitable for neural stem cell (NSC) function. There were more neuronal cells present on the hydrogel surface incorporating IKVAV peptides compared to the pure PLEOF hydrogel surface [117]. The large number of cells on the IKVAV-PLEOF hydrogel were mostly viable following 7 days of incubation. In vivo biocompatibility was also tested, whereby IKVAV-PLEOF hydrogels were successfully integrated into subcutaneous rat tissue and the hydrogel was responsible for increased blood vessel formation [117]. The surface of the IKVAV-PLEOF hydrogel was also more conductive of cell adhesion, which was measured at 60–70% compared with 1–10% on the control PLEOF hydrogel [117]. The capacity for the hydrogels to promote NSC differentiation was evaluated, and more neurons were observed on the surface of IKVAV-PLEOF. Through this work, it is clear that the IKVAV-PLEOF hydrogel can successfully imitate the ECM through its biocompatibility with cells and tissues, along with the ability to stimulate NSC differentiation. These stem cell laden hydrogels may have applications in regenerative spinal cord injury for neural tissue engineering [117].

Sun et al. [118] investigated the modification of IKVAV onto a silk fibroin-based hydrogel for use as a brain tissue engineering scaffold [118]. NSCs were viable in both the silk fibroin hydrogel modified with IKVAV and the unmodified hydrogel, but the IKVAV modified hydrogel showed a higher viable cell count overall [118]. Cell proliferation data further supported this result, where IKVAV-modified hydrogels showed increased cell proliferation compared to the unmodified hydrogel. The neuronal differentiation potential of both hydrogels was also investigated, with more cells in the IKVAV modified hydrogels being positive for the neuronal differentiation markers βIII-tubulin and microtubule-associated protein 2 (MAP-2), compared with those in unmodified hydrogels [118]. These results suggest that silk fibroin hydrogels modified with IKVAV could be used as biomimetic scaffolds for brain tissue engineering applications, following injuries such as trauma and stroke [118].

### 5.7. Laminin: YIGSR Motif

The pentapeptide sequence YIGSR is found in the β1 chain of laminin and has been identified as a cell adhesion site, which can bind to the 67 kDa laminin adhesion receptor [119]. The YIGSR peptide was first found between residues 929–933 on the β1 chain [120]. YIGSR-NH_2_ has been used to modify a poly (N,N-diethylacrylamide) (PDEAAm) hydrogel scaffold [121]. After initially seeding human embryonic stem cells (hESCs) onto the hydrogels, the PDEAAm gels did not possess cytotoxic traits and could stimulate increased cell attachment and proliferation compared with an unmodified hydrogel control, where cell attachment was significantly decreased. This confirmed the significance of the pentapeptide motif YIGSR in creating a biomimetic surface for the culture of hESCs [121].

In a study by Brandl et al. [122], degradable and non-degradable PEG hydrogels modified with YIGSR could promote a biomimetic environment suitable for adipocyte differentiation [122]. Although studies have shown that YIGSR can have a profound effect on cell attachment and proliferation, this work demonstrated that there were no significant differences in cell number between hydrogels modified with YIGSR, and hydrogels containing no YIGSR [122]. Significant differences were noted for adipocyte differentiation depending on the presence or absence of YIGSR; triglyceride accumulation in degradable hydrogels decreased from 400 µg per 100,000 cells in the presence of YIGSR to 260 µg per 100,000 cells in the absence of YIGSR [122]. Functionalising hydrogels with integrin-binding motifs such as YIGSR therefore support the accumulation of lipids within mature, differentiated adipocytes [122]. Future applications could involve further optimization of cell culture conditions to produce mature fat pads. The hydrogel-cell mixture could also be injected directly into a soft tissue defect, where in vivo crosslinking will produce functionally active tissue substitutes [122].

### 5.8. Elastin: GRKRK Motif

Tropoelastin contains a C-terminal pentapeptide sequence GRKRK [123], between residues 782–786. The adhesive properties of the synthetic peptide GRKRK were confirmed by adding GRKRK to cells already adhered to tropoelastin, which reduced cell adhesion from 50% to 16% [123]. Cation dependency was also used to determine if GRKRK adhesion was integrin mediated. Without metal ions, the GRKRK motif supported only 5% adhesion; however, in the presence of Mn^2+^, 58% cell adhesion was observed [123]. Although GRKRK has been identified as a key integrin-binding adhesive site within tropoelastin, to the best of our knowledge, there have been no studies which incorporate this adhesion motif within hydrogels.

### 5.9. The Effect of Functionalising Hydrogels with ECM-Derived Peptide Motifs on Gelation

The process of gelation begins with crosslinking polymerisation, during which gelation will occur at a specific point. At this time, often termed the “gel point”, visible gel formation can be seen, where a network of polymer molecules have been crosslinked to one another [124]. Several factors are well known to promote changes in hydrogel gelation. These include molecular weight, hydrophobicity, degree of crosslinking, gel nanostructure and temperature [125]. In the context of this review, it is important to consider how hydrogel gelation properties may be affected by the addition of ECM-derived peptide sequences.

An RGD-functionalised hydrogel was prepared from four polymers: PAA (polyacrylic acid), carbomer, agarose and PEG [126]. Through analysis of the swelling equilibrium ratio (an indicator of the ability to absorb and maintain large volumes of water), the analysed hydrogels demonstrated rapid swelling characteristics and the potential to reach swelling equilibrium within 1 h. Swelling equilibrium values of the functionalised hydrogels were also found to be similar to hydrogels without RGD modification, suggesting that the presence of the RGD tripeptide did not hinder the process of gelation. Additionally, the rheological properties of the hydrogel were not affected by RGD functionalisation, with the integrity of injectable properties such as viscoelasticity being maintained [126]. Mauri et al. [127] obtained similar data, where RGD functionalised and unfunctionalised hydrogels possessed fast swelling kinetics (swelling equilibrium was established within 30 min). The RGD peptide was integrated within the polymer network and did not impede gelation [127].

Some reports suggest that the type of peptide sequence used to functionalise hydrogels can vary the effects on gelation. In a study by Anderson et al. [128] peptide amphiphile (PA) gels were functionalised with a range of peptide sequences derived from the ECM. Following self-assembly, moderately stable gels were formed with PA-RGDS, while PA-DGEA and PA-YIGSR produced viscous solutions and PA-S demonstrated the strongest gel consistency. The findings suggest that the application of different peptide sequences to modify hydrogels may alter mechanical properties, and that variations will be seen depending on the sequence used [128].

### 5.10. The Synergy Effect

It is important to consider that although short peptide sequences such as LDV or RGD play a vital role for integrin binding, there are other synergistic sequences which add to their effect in a collaborative fashion [129]. Perhaps this synergistic effect should be considered more comprehensively in the context of biomimetic materials, by using multiple peptide motifs in tandem to functionalise materials of interest, including hydrogels.

## 6. Hydrogels Functionalised with Multiple Adhesion Motifs

A combination of short peptide motifs may be suitable for creating biomimetic scaffolds, where differing concentrations of bioactive motifs can be used for functionalisation. This may help to create a biomimetic environment which more accurately represents what cells would experience in the native ECM, because in vivo, cells are exposed simultaneously to an array of ligand types, at various concentration gradients [130].

PEG hydrogels have been modified with RGDS, IKVAV and YIGSR to create an environment capable of promoting nerve regeneration [130]. When all three bioactive ligands were used in combination, an additive effect on neurite extension was seen compared to RGDS alone, whereas all other pairwise combinations demonstrated an inhibitory effect or no effect whatsoever [130]. In a 2020 study, biocompatible hydrogels with improved cytotoxicity were produced using a combination of IKVAV and YIGSR peptides [131]. Rong et al. [95] have also used a combination of RGD and N-cadherin to enhance differentiation potential using PLG hydrogels. Glycosaminoglycan production was shown to be significantly greater in dual peptide modified PLG, compared to PLG and PLG/RGD hydrogels [95]. It is evident that to create a biomimetic material, there may be advantages to seeding cells onto materials which allow simultaneous cell interaction with multiple bioactive ligands [130], but the combinations and number of ligands can show varying outcomes.

Alongside the use of adhesion motifs in combination, some groups have reported that varying the density/concentration of adhesion motifs can affect cell behaviour. Lee and Lee. [132] reported that dual functionalising hydrogels with the ligands RGD and YIGSR had a positive effect on PC12 adhesion and proliferation [132]. Good cell adhesion was observed on hydrogels containing equal concentrations of RGD and YIGSR. However, PC12 proliferation significantly increased as the density of RGD increased but YIGSR density remained unchanged [132]. Additionally, increasing YIGSR density in dual peptide hydrogels also had a positive effect on the production of the neuronal maker growth-associated protein 43 (GAP43), which in suggested promotion of neuronal differentiation [132]. Fraser and Benoit, [133] cultured periodontal ligament stem cells (PDLSCs) in dual functionalised hydrogels containing RGD and GFOGER peptides to mimic the periodontal ECM [133]. More than 90% viability was maintained within all hydrogels tested [133]. ALP production and mineralisation were both enhanced by the presence of RGD and GFOGER when combined at varying concentrations; when high levels of RGD were paired with low-moderate levels of GFOGER, ALP activity increased by over half. Similarly, mineralisation increased by around 5-fold using moderate levels of RGD and high levels of GFOGER [133]. Dvořáková et al. [134] developed and characterised a synthetic polymer hydrogel poly (N^5^-(2-hydroxyethyl)-l-glutamine), modified with the integrin-binding motif RGD (PHEG-Tyr-RGD). Following cell encapsulation in PHEG-Tyr-RGD (6 mM) for 14 days, the cells demonstrated a normal MSC phenotype with a ‘spread’ morphology. As the concentration of RGD was increased, cell–matrix and cell–cell interactions increased. Overall, this polymer hydrogel was found to be biocompatible, facilitating the adhesion and proliferation of MSCs, with interactions between the cells and the matrix itself influenced by RGD concentration [134].

## 7. Conclusions

Evidence for the improved biocompatibility of a range of hydrogels functionalised via ECM adhesion motifs is abundant (Table 3). However, focus now needs to be given to more complex multicomponent hydrogels consisting of self-assembling peptides, rather than a fully synthetic or natural backbone. Such hydrogels could be functionalised with one or more ECM adhesion motifs, relevant to the anatomical site for tissue engineering. Multicomponent biocompatible hydrogels represent an emerging class of novel hydrogels that merit future study to expedite the translation of biomimetic hydrogels to therapeutic use.

## Figures and Tables

**Table 1:** Primary collagen types classified according to class, type, genes and higher-order struc- tural assembly [54].

Class	Type	Genes Encoding Proteins Common in Collagen	Description
Fibril-forming collagens	I II III V XI XXIV XXVII	*COL1A1* and *COL1A2* *COL2A1* *COL3A1* *COL5A1*, *COL5A2* and *COL5A3* *COL11A1*, *COL11A2*, *COL11A3* *COL24A1* *COL27A1*	Quarter-stagger arrangement of fibrils which are packed into stromal tissues such as bone and tendons
Network-forming collagens	IV	*COL4A1, COL4A2, COL4A3*, *COL4A4, COL4A5, COL4A6*	Network structure composed of laminins and basement membrane proteins
Fibril-associated collagens	IX XII XIV XVI XIX XX XXI XXII	*COL9A1, COL9A2, COL9A3* *COL12A1* *COL14A1* *COL16A1* *COL19A1* *COL20A1* *COL21A1* *COL22A1*	Fibril-associated collagens with interrupted triple helices (FACIT); molecular bridges associated with type I and type II collagen fibrils

**Table 2:** The four families of proteoglycans are primarily classified according to their location, gene/protein homology and the presence of particular protein units. This table aims to describe the proteoglycan classification system in a simplified form, highlighting the importance of cellular and subcellular location as well as the addition of specific glycosaminoglycan chains, which ultimately depends on the proteoglycan in question. Adapted from [66].

Location	Example	Gene Symbol	Glycosaminoglycan Chain
Intracellular	Serglycin	*SRGN*	Heparin
Cell surface	Betaglycan Syndecan	*TGFBR3* *SDC*	Chondroitin sulfate/Heparan sulfate Heparan sulfate
Pericellular	Agrin Collagen XV	*AGRN* *COL15A1*	Heparan sulfate Chondroitin sulfate/Heparan sulfate
Extracellular	Biglycan Decorin	*BGN* *DCN*	Chondroitin sulfate Dermatan sulfate

**Table 3:** A collection of ECM peptides based on studies that have been used to functionalise hydrogels and their cellular applications.

ECM Peptide	Hydrogel	Cell Application	Cell Type	Reference
GFOGER	PEG	Proliferation Differentiation	MSCs	[100]
		Viability Differentiation	MSCs	[101]
		Viability and Signalling Differentiation	hBMSCs	[102]
	PEG-PCL	Adhesion Spreading Regeneration	BMSCs	[103]
DGEA	Alginate	Adhesion Viability Differentiation	MSCs	[106]
P15	Hyaluronate (with ABM)	Attachment Migration Viability Differentiation	Osteoblast- like HOS cells	[111]
RGD	PEG	Viability Proliferation Differentiation	hPDCs	[91]
		Viability	MSCs	[92]
		Proliferation Differentiation	MSCs	[100]
	Chitosan	Attachment Proliferation Spreading Differentiation	BMSCs	[93]
	Vitrogel	Viability Differentiation Adhesion Spreading	hASCs	[97]
	Alginate	Viability Spreading Proliferation	HUVECs	[94]
	Polypeptide	Viability Spreading Proliferation	BMSCs	[95]
	Poly organo- phosphazene	Viability Spreading Proliferation	MSCs	[96]
	Hyaluronic acid/Pectin	Differentiation	Chondrocytes	[98]
	PHEG	Adhesion Spreading Proliferation Cell–cell and cell–matrix interactions	MSCs	[134]
LDV	Peptide	Viability Spreading	Fibroblasts	[114]
IKVAV	PLEOF	Viability Adhesion Differentiation	Neural stem cells	[117]
	Silk fibroin	Viability Proliferation Differentiation	Neural stem cells	[118]
YIGSR	PEG	Differentiation Attachment Proliferation	Adipocytes	[122]
RGDS, IKVAV YIGSR	PEG	Neurite extension	PC12 cells	[130]
IKVAV, YIGSR	Peptide	Improved cytotoxicity	C6 glial cells	[131]
			SHSY5Y neuro- blastoma cells	
RGD, N-cadherin	PLG	Differentiation	BMSCs	[95]
RGD, YIGSR	Alginate	Adhesion Proliferation Differentiation	PC12 cells	[132]
RGD, GFOGER	PEG	Viability Mineralisation	Periodontal ligament stem cells	[133]

## Data Availability

Not applicable.
